# Mandibular Advancement Devices in Obstructive Sleep Apnea and Its Effects on the Cardiovascular System: A Comprehensive Literature Review

**DOI:** 10.3390/jcm13226757

**Published:** 2024-11-10

**Authors:** Agnieszka Polecka, Jakub Nawrocki, Maria Alejandra Pulido, Ewa Olszewska

**Affiliations:** 1 Department of Cardiology and Internal Medicine with Cardiac Intensive Care Unit, Doctoral School of the Medical University of Bialystok, Medical University of Bialystok, 15-089 Bialystok, Poland; 2 Clinic of Orthodontics, Wroclaw University Dental Center, Krakowska 26, 50-425 Wroclaw, Poland; kuba.nawrocki123@gmail.com; 3 Whipps Cross & Royal London Hospital, Barts Trust NHS, London E11 1NR, UK; mariale.pulido@gmail.com; 4 Sleep Apnea Surgery Center, Department of Otolaryngology, Medical University of Bialystok, 15-089 Bialystok, Poland

**Keywords:** obstructive sleep apnea, oxidative stress, inflammation, endothelial damage, oral appliances, mandibular advancement devices, continuous positive airway pressure, sleep surgery, cardiovascular system, heart, sleep

## Abstract

Background: Obstructive sleep apnea syndrome (OSA) is a chronic inflammatory disease characterized by endothelial dysfunction and cardiovascular complications. Continuous positive airway pressure (CPAP) is the standard treatment, hence poor adherence has prompted interest in mandibular advancement devices (MAD) as an alternative. This comprehensive review aimed to explore the effects of MAD therapy on oxidative stress, inflammation, endothelial function, and its impact on the cardiovascular risk in OSA patients. Results: MAD therapy significantly reduces the apnea-hypopnea index (AHI), improves serum nitric oxide (NOx) concentrations, reduces oxidative stress markers, and enhances endothelial function. Animal studies indicated that MAD reduces myocardial fibrosis and attenuates inflammatory markers. While both CPAP and MADs improve endothelial function and heart rate variability, CPAP is more effective in reducing OSA severity. Nevertheless, MAD has higher compliance, contributing to its positive impact on cardiovascular function. Moreover, CPAP and MADs have similar effectiveness in reducing cardiovascular risk. Conclusions: MAD therapy is an effective alternative to CPAP, particularly for patients with mild to moderate OSA as well as those intolerant to CPAP. It offers significant improvements in endothelial function and oxidative stress. Further studies are needed to assess MAD therapy in comprehensive OSA management.

## 1. Introduction

Obstructive sleep apnea (OSA), affecting approximately 9–38% of the adult population, is a sleep disorder characterized by repeated episodes of complete (apnea) or partial (hypopnea) upper airway obstruction during sleep with preserved respiratory muscle effort [1]. The severity of OSA is classified into three categories—mild (AHI 5—14.99), moderate (AHI 15—29.99), and severe (AHI ≥ 30)—based on the apnea-hypopnea index (AHI) which indicates the number of respiratory events (apneas and hypopneas) per hour of sleep [2]. It leads to intermittent hypoxia (IH), disrupted sleep architecture, and significant cardiovascular, metabolic, and neurocognitive consequences [3,4].

OSA is closely linked to endothelial dysfunction, a crucial factor in the development of cardiovascular complications. The IH observed in OSA individuals initiates a cascade of generating reactive oxygen species (ROS), systemic inflammation, and sympathetic activation. These mechanisms disrupt endothelial homeostasis, impairing the production of nitric oxide (NO) and other vasoactive mediators critical for maintaining vascular tone and promoting vasodilation. As a result, the endothelium becomes prone to vasoconstriction, increased vascular smooth muscle proliferation, hypercoagulability, and ultimately, thrombosis [5].

The endothelium, a heterogeneous tissue, plays a central role in regulating vascular contractility, cell growth, and the permeability of the vascular wall. Its ability to produce growth factors and vasoactive substances (i.e., NO) varies across different vascular beds, contributing to hypertension, coronary artery disease, and cerebrovascular disorders [6].

OSA can be managed through a variety of treatment approaches, depending on the severity of the condition and patient-specific factors. Common interventions include lifestyle changes (including weight reduction, smoking cessation, sleep hygiene, alcohol avoidance), continuous positive airway pressure (CPAP) therapy, upper airway surgeries, and myofunctional therapy [7,8]. Moreover, CPAP is the most widely considered gold standard for OSA treatment [9]. It prevents airway collapse during sleep and improves endothelial function by reducing the hypoxic burden and restoring vascular homeostasis, effectively reducing cardiovascular risk. However, despite its proven efficacy, adherence to CPAP can be challenging due to discomfort, claustrophobia, or mask intolerance, which often leads to poor compliance or discontinuation of treatment [10].

Mandibular advancement devices (MAD) offer an alternative therapy, particularly for patients with anatomical obstructions in the upper airway located in the oropharynx. Various types of MAD are available, including mono-block and dual-block designs. Mono-block devices are made as a single piece, while dual-block devices consist of two separate parts, allowing for adjustable jaw positioning. These devices work by repositioning the mandible and tongue forward, increasing the upper airway volume and reducing the likelihood of airway collapse [11]. Moreover, MAD may enhance the stiffness of the pharyngeal walls, improving airflow through the soft palate area. Consequently, MAD is recommended as a primary alternative for individuals with mild to moderate OSA, where CPAP might be poorly tolerated [12,13].

## 2. Materials

We searched PubMed, Scopus Library, Cochrane and Google Scholar for case-control studies and randomized control trials (RCTs) concerning the effectiveness of MADs in OSA and its cardiovascular effects. The search was performed using the words “obstructive sleep apnea”, “mandibular advancement device”, “mandibular advancement splint”, “oral appliances”, “oxidative stress”, “endothelial functions” and “cardiovascular disease” in different combinations by using AND and/or OR terms. The search results were exported to the Mendeley reference manager for the records’ initial title and abstract screening. Duplicate articles were removed. The literature search was performed between 1 September 2024 and 1 October 2024. Two authors (A.P. and J.N.) independently performed the literature search and evaluated articles for inclusion. Discrepancies, if any, were resolved through discussion. During the initial screening of titles and abstracts, the retrieved studies had to meet the following inclusion criteria in full-text eligibility assessment: (1) randomized control trials or case-control studies; (2) papers concerning adult human participants and animal models diagnosed with OSA (AHI ≥ 5), (3) studies evaluating a combination of OSA and cardiological parameters with oxidative stress or endothelial function, or inflammation; (4) clearly defined experimental and control groups. Exclusion criteria were: (1) studies in other than English language, (2) studies on pediatric population (i.e., age < 18 years), (3) article review, letter, poster, conference summary or editorial. After the initial screening, two investigators (A.P. and J.N.) retrieved and independently assessed full-text manuscripts. Any disagreements were resolved through discussion or consultation with a third author (E.O.). A total of 17 studies met the inclusion criteria and were included in the qualitative assessment.

### 2.1. Effects of MAD on Oxidative Stress

Itzhaki et al. conducted a study on 16 adult patients and assessed the effect of long-term modified Herbst mandibular advancement splint (MAS) treatment on OSA and oxidative stress markers. The study measured the concentration of lipid peroxidation for thiobarbituric acid-reactive substances (TBARS, malondialdehyde (MDA)) and peroxide (PD). TBARS concentration decreased from 18.8 ± 6.2 nmol malondialdehyde (MDA)/mL before treatment to 15.8 ± 3.9 MDA/mL after 3 months (*p* = 0.09) and 15.5 ± 3.2 nmol MDA/mL after 1 year (*p* < 0.05). The changes in TBARS concentrations at both the 3-month and 1-year evaluations showed a positive correlation with the corresponding changes in AHI (r = 0.59 and 0.51, respectively; *p* < 0.05). The untreated control group showed no changes. Moreover, AHI decreased significantly from 29.7 ± 18.5 before treatment to 17.7 ± 11.1 after 3 months of treatment and 19.6 ± 11.5 after 1 year of treatment (*p* < 0.005 for both). The mean Epworth Sleepiness Scale score decreased significantly from 12.4 ± 6.0 before treatment to 10.2 ± 6.6 after 3 months of treatment and 7.8 ± 3.8 after 1 year of treatment (*p* < 0.001 for both) [14].

Lin et al. conducted a study on 30 patients with moderate-to-severe OSA who used MAD therapy and 15 healthy controls to assess the effects of mandibular advancement devices on serum nitric oxide derivatives (NOx), determined via ELISA from peripheral blood samples. All participants underwent sleep studies, which were repeated 2 months after MAD treatment in the OSA group. Before MAD treatment, patients with OSA had significantly lower serum NOx levels compared to healthy controls. In 19 of the 30 OSA patients who responded to MAD therapy, serum NOx levels increased from 11.8 ± 5.8 μM pre-MADs to 22.7 ± 4.9 μM post-MAD. However, the 11 non-responders showed no significant improvement in serum NOx concentration [15].

Liu et al. conducted a study to assess the effect of MAD treatment on myocardial health in OSA-induced rabbits. Thirty New Zealand rabbits were divided into three groups: a control group, an OSA group, and a MAD group. To induce obstruction of upper airways, hydrophilic polyacrylamide gel was injected into the soft palates. In the MAD group, a mandibular advancement device was applied after OSA was established. The animals were placed in a supine position to sleep for 4–6 h per day over eight weeks. Plasma concentrations of endothelin-1 (ET-1), angiotensin II (Ang II), and the expression of ET-1 mRNA in heart tissue were assessed. Elevated plasma concentrations of ET-1 and Ang II, as well as increased ET-1 mRNA expression in cardiac tissue were observed in the OSA group compared to the control and MAD groups. Additionally, blood oxygen saturation was inversely correlated with ET-1 and Ang II concentrations [16].

Dal-Fabbro et al. performed a randomized, crossover, single-blind, controlled trial on 29 adults with moderate-to-severe OSA individuals who underwent treatment with MADs, CPAP, and a placebo oral appliance. Assessments included oxidative stress markers—malondialdehyde, catalase, superoxide dismutase, vitamins C, E, B6, B12, folate, homocysteine, and uric acid at baseline and after one month of each treatment. Compliance was tracked using diaries and a pressure-time meter for CPAP. Both MADs and CPAP reduced the AHI (*p* < 0.05) and Epworth Sleepiness Scale, with CPAP having a greater impact (*p* < 0.05). There was a significant reduction in arousal index (*p* < 0.05) after both CPAP and MADs compared with baseline. Catalase activity decreased significantly after MAD treatment. The analysis of lipid peroxidation products, measured via thiobarbituric acid-reactive substance (TBARS) evaluation, showed no significant differences between treatments (*p* < 0.53). Similarly, no significant differences were observed in erythrocyte superoxide dismutase activity (*p* < 0.51) or uric acid levels (*p* < 0.43). Erythrocyte catalase activity decreased significantly (*p* < 0.01) following MAD treatment compared to baseline (*p* < 0.05). Additionally, vitamin C concentrations increased significantly (*p* < 0.01) after both CPAP and MAD treatment, compared to baseline (*p* < 0.05). No significant changes were found in homocysteine, folate, or vitamins B12 and E (*p* < 0.05), while vitamin B6 concentrations significantly increased (*p* < 0.001) after all treatment phases compared to baseline (*p* < 0.05). Compliance was higher with MAD than CPAP [17].

The summarized data of chosen studies is presented in Table 1.

### 2.2. Effects of MAD on Inflammation

When analyzing data on animal studies, the study performed by Kang et al. aimed to explore the role of nuclear factor-kappa B (NF-κB) in cardiac structural changes in OSA rabbits treated with MADs [18]. Eighteen male New Zealand white rabbits were randomly assigned to three groups: control, OSA, and MAD. To induce OSA in the OSA and MAD groups, hyaluronate gel was injected into the rabbits’ soft palates. Subsequently, levels of NF-κB, Interleukin 6 (IL-6), Interleukin 10 (IL-10), and the proportion of myocardial fibrosis (MF) were measured at baseline and 8 weeks follow-up. The results showed that NF-κB, IL-6, and IL-10 concentrations were higher in the OSA group compared to the controls (*p* < 0.05), resulting in an increased accumulation of collagen fibers (the levels of cardiac collagen fibers Type I/Type III in OSA group increased significantly compared with controls (*p* < 0.05)). The AHI was positively correlated with inflammatory factors. In contrast, no significant differences were observed between the MAD-treated and control groups. Additionally, the increased AHI in the OSA rabbit group, which correlated negatively with reduced levels of medium oxygen saturation (MOS) and lowest oxygen saturation (LOS), was corrected by MAD [18].

Niżankowska-Jędrzejczyk et al. conducted a study examining the effects of MAD therapy in patients with mild-to-moderate OSA. The authors analyzed both pro-inflammatory and anti-inflammatory cytokine concentrations, revealing increased concentration of IL-1β (pro-inflammatory) and IL-10 (anti-inflammatory) in OSA patients. MAD treatment led to significant reductions in the abovementioned cytokines, indicating a modulation of the inflammatory response [19].

The summarized data of chosen studies is presented in Table 2.

### 2.3. Effects of MAD on Endothelial Function

A study conducted by Itzhaki et al. on 16 adult patients that had been mentioned in Section 2.1. assessed the effect of long-term modified MAS treatment also on endothelium functions (EF) and OSA. The authors performed the RH-PAT test, which measures endothelial function using finger plethysmography. The device records pulse wave amplitude in the test and controls fingers across three stages: baseline, occlusion, and post-occlusion. The RH-PAT index is calculated by comparing the pulse amplitude before and after occlusion, normalized to the control hand. EF improved significantly from 1.77 ± 0.4 before treatment to 2.1 ± 0.4 after 3 months (*p* < 0.05), and 2.0 ± 0.3 after 1 year (*p* = 0.055), similar to the reference group [14].

Moreover, a study by Lin et al. evaluated the effects of MAD on endothelial function by endothelium-dependent flow-mediated dilation (FMD) in patients with OSA. They enrolled 30 patients with moderate-to-severe obstructive sleep apnea who used MAD therapy and 15 healthy controls. FMD was evaluated using high-resolution B-mode ultrasonography. Before MAD treatment, OSA patients had significantly lower FMD compared to healthy controls. In 19 out of the 30 OSA patients who responded to MAD therapy (defined as AHI < 5 or AHI >5 and >50% decrease in AHI), FMD improved from 5.9 ± 4.6 pre-MADs to 10.5 ± 4.8 post-MADs. However, the 11 non-responders (AHI > 5 and < 50% decrease in AHI) showed no significant improvement in FMD after the treatment [15].

A randomized controlled trial by Gagnadoux et al. involving 150 patients with severe OSA (86% male; mean age 54 years) assigned to receive either MAD treatment or a sham device. The results demonstrated that the use of MAD significantly reduced AHI (*p* < 0.001), micro-arousal index (*p* = 0.008), and symptoms including snoring, fatigue, and sleepiness (*p* < 0.001) compared to the sham device. However, no significant improvements were observed in endothelial function or blood pressure outcomes between the two groups. Objective compliance was higher in the effective MAD group, averaging 6.6 h per night compared to 5.6 h per night in the sham device group (*p* = 0.006) [20].

A study conducted by Trzepizur et al. aimed to investigate the impact of 2 months of CPAP and MAD treatment on microvascular endothelial function (MVEF) in OSA patients. Microvascular reactivity was assessed using laser Doppler flowmetry with acetylcholine (Ach) and sodium nitroprusside (SNP) iontophoresis in 24 OSA patients and 9 control subjects. In a randomized crossover design, 12 OSA patients were re-evaluated on microvascular reactivity after 2 months of CPAP and MAD treatment. The study revealed that Ach-induced vasodilation was significantly lower in OSA patients compared to controls and negatively correlated with AHI (r = −0.49, *p* < 0.025), and nocturnal oxygen desaturation (r = −0.63, *p* < 0.002). Both CPAP and MAD treatments led to significant improvements in Ach-induced vasodilation, correlated with the reduction in nocturnal oxygen desaturation (r = 0.48, *p* = 0.016) [21].

The summarized data of chosen studies is presented in Table 3. 

### 2.4. Effects of MAD on the Cardiovascular System

#### 2.4.1. Heart Rate Variability 

Kim et al. investigated the effects of MAD on nocturnal heart rate variability (HRV) in patients with OSA. The study on 58 adult OSA patients treated with MAD therapy retrospectively reviewed anthropometric data, questionnaire results, and HRV parameters (both time-domain and frequency-domain methods). All patients underwent baseline polysomnography and a follow-up after 3 months of treatment. Significant changes in HRV metrics, including the average normal-to-normal (NN) interval, the standard deviation of the NN interval, low-frequency power (LFnu), and high-frequency power (HFnu) were observed with MAD therapy. Based on treatment success (defined as a reduction in the AHI of more than 50% and an AHI below 20), thirty-four patients were classified as responders, while 24 were non-responders. Baseline characteristics between the groups were similar, except for a higher body mass index (BMI) and lower LOS in the non-response group. Subgroup analysis revealed that in the response group, the average NN interval and HFnu significantly increased, while total power (TP), very low frequency (VLF), low frequency (LF), LF/HF ratio, and LFnu decreased compared to baseline. In contrast, no significant HRV changes were detected in the non-response group. After adjusting for age, sex, and BMI, the response group exhibited significant improvements in TP and LF relative to the non-response group [22].

Glos et al. conducted a randomized, two-period crossover trial to compare the effects of MAD concurrently with CPAP on daytime cardiac autonomic modulation. The authors enrolled 40 OSA patients who underwent treatment with MAD (*n* = 19) and CPAP (*n* = 21) for 12 weeks each. At baseline and following each treatment phase, polysomnography was conducted, and a daytime cardiac autonomic function test was administered. This test measured HRV, continuous blood pressure (BP), and baroreceptor sensitivity (BRS) during spontaneous breathing and controlled breathing at 6, 12, and 15 breaths per minute. The study revealed that both MAD and CPAP therapies effectively reduced apneas and hypopneas, though CPAP had a more pronounced effect. For all four controlled breathing conditions, three-minute averages of continuous diastolic BP were significantly lowered with both therapies. MAD therapy, in particular, led to selective increases in high-frequency HRV values, indicating improved autonomic function. No significant changes in BRS were observed for either treatment. Both MAD and CPAP therapies provide similar improvements in daytime cardiac autonomic function, and CPAP was more effective at eliminating respiratory events [23].

Lee et al. conducted a study to compare the effectiveness of sleep surgery and MADs on OSA and cardiovascular outcomes. Thirty adult subjects were enrolled in the sleep surgery group or the MAD group, matched for sex, BMI, and baseline AHI. The effectiveness of treatment was assessed with polysomnography, sleep quality questionnaires, and HRV time- and frequency-domain parameters at baseline and 3-month follow-up. The results demonstrated significant improvements in both polysomnographic and sleep quality questionnaire parameters in both treatment groups. In time-domain HRV analysis, the average normal-to-normal intervals increased significantly in both the surgery group (from 942.2 ± 140.8 to 994.6 ± 143.1, *p* = 0.008) and the MAD group (from 901.1 ± 131.7 to 953.7 ± 123.1, *p* = 0.002). In the sleep surgery group, low-frequency (LF) power decreased significantly (*p* = 0.012), while high-frequency (HF) power remained unchanged in both groups. The LF/HF ratio decreased in both groups (2.9 ± 1.8 to 2.3 ± 1.7, *p* = 0.017 for surgery; 3.0 ± 1.8 to 2.4 ± 1.4, *p* = 0.025 for MAD), and normalized high-frequency power increased significantly in both groups (31.0 ± 13.2 to 36.8 ± 13.7, *p* = 0.009 for surgery; 29.1 ± 10.7 to 33.7 ± 12.5, *p* = 0.024 for MADs). However, no significant differences in HRV parameter changes were found between the two groups after adjusting for age, BMI, and AHI. Additionally, the authors suggested that observed HRV changes might indicate that both therapies could reduce OSA-related cardiovascular mortality [24].

Dal-Fabbro et al. performed a randomized, crossover, single-blind, controlled trial on 29 adults with moderate-to-severe OSA participants who underwent treatment with MADs, CPAP, and a placebo oral appliance. In the context of HRV analysis, there was a reduction in the absolute value of total power (at night) with both MAD and CPAP groups compared to placebo (*p* < 0.05, *p* < 0.05, respectively), and MADs improved sleep autonomic modulation compared to baseline (*p* < 0.05) [17].

Ucak et al. conducted a study evaluating the impact of MAD therapy on cardiac autonomic function in 101 adult OSA patients. The authors indicated a positive correlation between markers of enhanced cardiac autonomic adaptability and effective MAD treatment demonstrated by lengthened NN intervals and the increase in pNN50 (percentage of absolute differences in successive NN values greater than 50 ms). Specifically, for every one-unit reduction in the AHI, a corresponding increase of 2.21 units in the NN interval length was observed, indicating improved parasympathetic activation. However, the study did not find correlations between changes in AHI and HRV frequency domain measures [25].

The summarized data of chosen studies is presented in Table 4.

#### 2.4.2. Left Ventricle Structure and Function

Dieltjens et al. conducted a prospective trial aimed to assess the effects of MAD therapy on left ventricular (LV) structure and function over six months. At the baseline and six-month follow-up, participants underwent a home sleep study, 24-hour ambulatory blood pressure monitoring, and two-dimensional Doppler and tissue Doppler echocardiography to evaluate cardiac function. A total of 63 patients (average age 49, BMI 27.0, and baseline AHI of 11.7) completed the study. The results showed that blood pressure and LV function parameters did not change significantly with MAD therapy. However, the interventricular septum thickness, which was at the upper limit of normal at baseline, decreased significantly after six months of MAD therapy (11.1 mm to 10.6 mm, *p* = 0.03). This reduction in septal thickness was observed only in patients classified as responders to MAD therapy, while non-responders showed no significant change. Importantly, the decrease in interventricular septum thickness was not correlated with changes in blood pressure [26].

Hoekema et al. conducted a study on OSA patients without cardiovascular disease (CVD) to evaluate left ventricular structure and compared the effects of MAD therapy with CPAP therapy. The study involved 28 patients with moderate to severe OSA, 15 of them were randomized to receive MAD therapy, while 13 received CPAP therapy. The study revealed that among the 28 patients, 7 had left ventricular hypertrophy, 6 had left ventricular dilatation at baseline. No significant improvements in echocardiographic outcomes were observed after 2–3 months of treatment in either group [27].

Additionally, evidence from animal studies is available. Liu et al. conducted a study to assess the effect of MAD treatment on myocardial structure and function in OSA-induced rabbits. Thirty New Zealand animals were divided into three groups: a control group, an OSA group, and a MADs group. To induce obstruction of upper airways, hydrophilic polyacrylamide gel was injected into the soft palates. In the MAD group, a mandibular advancement device was applied after OSA was induced. The animals were placed in a supine position to sleep for 4–6 h per day over eight weeks. Echocardiography and histological changes were examined. After 3 months of treatment, in the controls, the myocardial fibers appeared intact and orderly. In contrast, the OSA group exhibited significant disruption of myocardial structure, including extensive collapse, fissuring, and disorganization of the cardiac muscle fibers. However, these structural abnormalities were not observed in the myocardium of MADs group [16].

The summarized data of chosen studies is presented in Table 5.

#### 2.4.3. NT-proBNP

Previously mentioned study by Hoekema et al. evaluated the amino-terminal fragment of pro-brain natriuretic peptide (NT-proBNP), and compared the effects of MAD therapy with CPAP therapy. After 2 to 3 months of treatment, NT-proBNP measurements were repeated. The study revealed that among the 28 patients, 3 had elevated NT-proBNP concentration at baseline. After treatment, the median NT-proBNP concentration improved significantly following MAD therapy, decreasing from 52 pg/mL to 22 pg/mL. In contrast, CPAP therapy did not result in a significant change in NT-proBNP levels, which increased from 31 pg/mL to 37 pg/mL [27].

The study conducted by Uniken Venema et al. on 54 moderate OSA patients (24 in the MAD group and 30 in the CPAP group) aimed to compare the cardiovascular effects of MAD therapy versus CPAP. Baseline and 12-month follow-up assessments were performed. Both treatments significantly reduced AHI, however, the reduction was significantly greater (*p* < 0.01) with CPAP (mean reduction AHI 19.1 ± 4.3) compared to MAD group (mean reduction AHI 10.3 ± 12.0). No correlation was found between NT-proBNP and AHI in either the MADs or CPAP groups. There was no significant difference in NT-proBNP concentrations between baseline and the 12-month follow-up in both groups [28].

The summarized data of chosen studies is presented in Table 6.

#### 2.4.4. Cardiovascular Events

Phillips et al. conducted a randomized crossover trial to assess cardiovascular outcomes after 1 month of optimal CPAP and MAD therapy in OSA participants. The measurements included 24-hour mean arterial blood pressure, arterial stiffness, and quality of life (Short Form-36), and were compared between treatments. A total of 126 patients with moderate-severe OSA were randomly assigned to the trial. Comparing both treatments, there was a significant reduction in AHI in the CPAP group (CPAP group: AHI 4.5 ± 6.6/h; MADs group: AHI 11.1 ± 12.1/h; *p* < 0.01). Reported compliance was higher on MAD (MAD group, 6.50 ± 1.3 h per night vs. CPAP group, 5.20 ± 2 h per night; *p* < 0.00001). The 24-hour mean arterial blood pressure was comparable between both groups (CPAP-MAD difference, 0.2 mm Hg [95% confidence interval, −0.7 to 1.1]), however, neither treatment demonstrated a significant reduction in blood pressure overall [29].

A randomized controlled trial by Anandam et al. compared the effectiveness of MAD and CPAP therapy in reducing cardiovascular mortality in patients with severe OSA. The study consisted of 562 participants with severe OSA (who received CPAP at baseline), and a control group (269 individuals without OSA). Three months after CPAP initiation, 57% (319 out of 562) non-adherent individuals were offered MAD therapy. Fatal cardiovascular events were defined as death from stroke, myocardial infarction, sudden cardiac arrest or cardiac arrhythmias. They concluded that both CPAP and MAD might have similar effectiveness in reducing the risk of fatal cardiovascular events in patients with severe OSA (hazard ratio 1.08 [95% CI: 0.55–1.74], *p* = 0.71). Additionally, the cumulative incidence of cardiovascular death was significantly higher in untreated patients compared to MADs (*p* = 0.047) or CPAP therapy (*p* < 0.001) [30]. 

The summarized data of chosen studies is presented in Table 7.

## 3. Discussion

Obstructive sleep apnea (OSA) is associated with increased cardiovascular mortality and morbidity, and severe OSA significantly increases the risk of fatal CV events [31,32]. The underlying mechanisms explaining associations between OSA and cardiovascular disease are not entirely delineated but might involve sustained sympathetic activation, intrathoracic pressure swings, and oxidative stress [33]. Additional factors such as coagulation disorders, endothelial damage, platelet activation, and elevated inflammatory mediators may contribute to CVD pathogenesis [34]. Moreover, evidence suggests CPAP treatment for sleep apnea reduces systolic blood pressure, improves left ventricular function, and reduces platelet activation, as well as decreasing the risk of fatal and nonfatal cardiovascular events, including myocardial infarction and coronary interventions with the need for coronary artery bypass surgery or percutaneous transluminal coronary angiography, or both [35,36]. Therefore, exploring alternative OSA treatments and their effects on key cardiovascular pathways like autonomic dysfunction, oxidative stress and endothelial disfunction may help reduce cardiovascular risks and enhance patient outcomes.

OSA can be managed through both invasive and non-invasive treatment options. Among non-invasive options, CPAP therapy and MADs are the most commonly utilized treatments, with CPAP as the first-line therapy due to its superior efficacy in reducing AHI. However, However, about 30–40% of patients become non-adherent to CPAP due to discomfort and noise [37]. Emerging research, including one by Francis et al., suggests MADs, while less effective in reducing AHI, can offer similar health outcomes compare to CPAP due to better patient adherence [10,16]. Moreover, Sutherland et al. found that 92% and 75% of participants adhered to MAD therapy at 6 months and 2 years, respectively, and 96.5% wished to continue at 5 years [38]. Nevertheless, a Consensus Statements among European Sleep Surgery Experts on Snoring and Obstructive Sleep Apnea conducted by Olszewska et al. proposed that several factors should be considered to approach patients’ needs, suggesting that in patients with mild OSA, a trial of MAD therapy might be beneficial before considering more invasive surgical treatments [11]. Future research should compare these treatments, highlighting patient-centered care and preferences [39].

Cardiovascular diseases remain the leading cause of mortality worldwide, with oxidative stress significantly contributing to various conditions [40]. Elevated oxidative stress disrupts the balance between reactive oxygen species (ROS) and antioxidants, which is essential for normal cellular function. While a basal concentration of ROS is necessary for cellular processes, excessive ROS damage DNA, lipids, and proteins, resulting in necrosis and apoptosis, reducing nitric oxide availability, and leading to vasoconstriction and arterial hypertension. Additionally, ROS disrupt myocardial calcium regulation, leading to arrhythmias, and contribute to cardiac remodeling by triggering hypertrophic signaling. Furthermore, ROS promote the development of atherosclerotic plaques [41].

Interestingly, even when examining the uvular mucosa in individuals with OSA, a tissue directly involved in upper airway obstruction, an oxidative/reductive imbalance was observed, and it was associated with the severity of the disease [42].

Several studies indicate that, beside the improvements in sleep study, MADs may reduce oxidative stress in OSA patients by enhancements in nitric oxide concentrations and reductions in lipid peroxidation, highlighting their potential to improve overall cardiovascular health [14,15,16,17]. Moreover, the reduction in oxidative stress was often positively correlated with AHI improvements, supporting MADs’ role in reducing OSA severity [14]. Additionally, MADs might enhance endothelial function by increasing NOx levels in patients who respond to therapy, potentially mitigating arterial blood pressure [15].

Sympathetic nervous system overactivity observed in OSA patients is effectively measured by the overproduction of endothelin-1 and Angiotensin II concentrations. ET-1, a potent vasoconstrictor, is produced by endothelial cells, while Ang II, a key component of the renin-angiotensin-aldosterone system (RAAS), regulates blood pressure, fluid balance, and vascular resistance. Renin converts angiotensinogen (produced by the liver) into angiotensin I, which is further converted into Ang II by angiotensin-converting enzyme (ACE), predominantly in the lungs. This leads to elevated circulating levels of Ang II. During episodes of airway obstruction hypoxia stimulates the production of ET-1 and increases renin release from the kidneys. Since renin is a critical enzyme in the RAAS, it leads to an increase in Ang II levels. Hypoxia also induces hypoxia-inducible factor (HIF), which may upregulate components of the RAAS pathway, enhancing Ang II production. Additionally, ROS stimulate the expression of endothelin-converting enzyme (ECE), which converts pro-endothelin to the active ET-1, thus elevating ET-1 levels in circulation. Inflammatory cytokines (e.g., interleukin-6) may upregulate renin and ACE expression, leading to more Ang II production. Ang II itself promotes oxidative stress and inflammation, creating a vicious cycle. ET-1 and Ang II are significant in cardiovascular alterations, Ang II prompts cardiac dysfunction, including hypertrophy, arrhythmia, and heart failure [43]. Additionally, chronic elevations in Ang II contribute to vascular remodeling (thickening of vessel walls) and increased vascular tone. This finding is significant in OSA because it leads to persistent increases in blood pressure, even during the daytime, which is often observed in OSA patients as a form of resistant hypertension. Moreover, the increased activation of Ang II leads to sodium and water retention via aldosterone release, which raises blood volume and contributes to hypertension. The fluid retention can also exacerbate OSA by increasing upper airway edema, making it more prone to collapse during sleep. 

Sleep fragmentation with frequent sleep arousals elevate sympathetic activity, increasing catecholamines (e.g., norepinephrine), which can directly stimulate the release of ET-1, and increases renin secretion from the kidneys, further elevating circulating levels of Ang II. Furthermore, negative intrathoracic pressure swings increase transmural pressure across the myocardium and increase afterload, which negatively impacts myocardial structure and function. In animal studies, MAD therapy has shown reductions in ET-1 and Ang II, improving left ventricular systolic and diastolic function [16]. These benefits are likely due to MADs’ ability to reopen the narrowed upper airway in OSA, improve blood oxygen saturation and prevent the elevation of intrathoracic pressure, left ventricular afterload, and sympathetic nerve activity. It helps mitigate the adverse impact of OSA on cardiac structure and function [11].

When comparing CPAP and MADs, both therapies significantly reduced AHI and improved sleep quality, with CPAP having a more pronounced effect. However, higher compliance with MADs was linked to reduced erythrocyte catalase activity and increased antioxidants, indicating reduced oxidative stress. These findings highlight the role of patient adherence in the therapeutic effectiveness of MADs and suggest that despite CPAP’s superior efficacy in reducing OSA severity, MADs might offer comparable benefits in reducing oxidative stress through improved compliance [17].

Repeated episodes of apneas and hypopneas cause chronic intermittent hypoxia followed by reoxygenation, activating pro-inflammatory transcription factors, like nuclear factor kappa B (NF-κB), activator protein-1, Interleukin-6 or Interleukin-10 [44,45]. These factors subsequently lead to the activation of inflammatory cells and an upregulation of pro-inflammatory mediators, contributing to oxidative stress, cytokine imbalance, systemic inflammation, increased coagulation, and endothelial dysfunction—all of which are linked to the development of atherosclerosis and the aging process [44,46]. As a result, individuals with OSA face a significantly elevated risk of cardiovascular diseases and metabolic disorders.

NF-κB, a key inflammatory mediator present in nearly all eukaryotic cells, and hypoxia-inducible factor 1 (HIF-1) are active in OSA individuals, with elevated levels of HIF-1α and NF-κB associated to cardiac inflammation, apoptosis, and fibrosis [47]. Clinical studies confirm elevated serum NF-κB and IL-6 in OSA patients, and animal studies showed increased NF-κB in the myocardium of mice with OSA [48,49]. Consequently, heightened activation of the NF-κB pathway could contribute to left ventricular remodeling and impaired cardiac function [47]. Conversely, MAD treatment normalized these parameters to levels comparable to the non-OSA individuals [16]. The positive correlation between AHI and inflammatory markers, underscores the potential of MADs to attenuate inflammation and fibrosis in the myocardium, protecting against structural heart damage.

In contrast, non-elevated CRP and IL-6 levels in OSA patients might be attributed to the milder OSA severity and lower body weight in the cohort suggesting that elevated CRP may be more related to obesity than OSA.However, elevated IL-1β, a marker of systemic inflammation, and fibrinogen in mild-to-moderate OSA patients, consistent with most reports, and MAD therapy significantly decreased IL-1β, indicating reduced systemic inflammation, similar to CPAP results. Interestingly, increased IL-10, an anti-inflammatory cytokine might reflect a compensatory response, though the 50% reduction after MAD therapy raises questions about its clinical relevance [19]. It remains unclear whether this decrease indicates a negative effect of MADs or a normalization of the cytokine profile during treatment, warranting further investigation.

Additionally, a systematic review by Priyadarshini et al. showed that while MADs effectively reduced the AHI in moderate to severe OSA, its impact on inflammatory and metabolic biomarkers was minimal and no overt CVD, despite high adherence and a significant OSA severity reduction. The systematic review indicates MADs improve polysomnographic metrics thus reducing the AHI alongside showing some effect on inflammatory biomarkers in OSA [50]. However, due to the lack of meta-analysis, statistically significant improvements could not be demonstrated.

OSA patients have shorter leukocyte telomere length and reduced SIRT1 protein levels compared to non-OSA individuals [51]. Telomere length in most human tissues declines with age, indicating the balance between oxidative stress and antioxidant defenses, and hypoxia accelerates this shortening [52]. Shorter telomeres are associated with a higher risk of age-related diseases, establishing telomere length as a biomarker for aging [53,54]. SIRT1 increase the expression of endothelial nitric oxide synthase, leading to enhanced nitric oxide production, thus preventing telomere shortening and protecting against vascular aging and cardiovascular diseases [55]. Following MAD treatment, these levels returned to normal, suggesting a potential beneficial effect of applied therapy [56].

Early signs of atherosclerosis, exhibited by endothelial dysfunction (ED), increased intima-media thickness, and decreased carotid diameter, significantly correlate with OSA severity [57,58]. MAD treatment has been shown to improve OSA symptoms and cardiovascular outcomes, including better endothelial function (EF) due to reduced oxidative stress and inflammation, as indicated by lower TBARS levels. By reopening the airway and improving oxygen saturation, MADs reduce the burden of hypoxia-reoxygenation cycles, which are known to damage the endothelium and reduce the frequency of ischemic events caused by repeated apneas and hypopneas, supporting the vascular health. MADs have also improved the microvascular EF in OSA, suggesting that impaired MVEF in OSA is related to disease severity, and can be mitigated by MADs [20]. While above-mentioned benefits make MADs a valuable alternative for patients who cannot tolerate CPAP, one particular study presents contrasting results, with a reduction in OSA-related symptoms, but no significant improvement in endothelial function [20]. These findings highlight the need for further research, as the variability in outcomes suggests that the impact of MADs on cardiovascular health may depend on individual patient factors.

Heart rate variability, which measures the balance between sympathetic and parasympathetic activity, has emerged as a crucial tool for assessing the effectiveness of OSA treatment, especially in the context of cardiovascular risk. It offers valuable insight into the autonomic dysregulation associated with OSA. After MAD therapy, increases in the average normal-to-normal interval and high-frequency power suggested improved parasympathetic activity. while decreases in low-frequency power and the LF/HF ratio indicated reduced sympathetic activity, which is crucial for cardiovascular risk reduction in OSA. Importantly, these changes were not observed in non-responders, underscoring the potential utility of HRV as an objective indicator of treatment effectiveness [22]. Interestingly, while CPAP was more effective in reducing apneas and hypopneas, MAD therapy selectively increased HF power, further emphasizing its beneficial effect on parasympathetic activity [23]. This suggests that MADs can restore autonomic balance in patients who tolerate them better than CPAP, offering cardiovascular protection through improved HRV. When comparing the impact of sleep surgery and MADs on HRV, significant improvements in NN intervals and reductions in the LF/HF ratio were observed in both groups [24]. The positive changes in HRV parameters imply that both treatments can reduce the cardiovascular risk associated with OSA.

Left ventricular hypertrophy, myocardial dysfunction, and overall cardiac remodeling significantly increase the risk of cardiovascular morbidity and mortality in OSA patients. While CPAP therapy partially reverses LV hypertrophy and improves left ventricle ejection fraction, the impact of MADs on LV remodeling remains unclear [59,60]. Studies show that although MAD therapy did not significantly alter blood pressure or LV function, it reduced interventricular septal thickness in MADs responders, indicating a reversal of LV hypertrophy [26]. This improvement, s, independent of blood pressure changes, suggests that OSA might directly contribute to LV hypertrophy, supporting the idea that effective OSA treatment, such as MADs, could reverse cardiac changes and reduce long-term cardiovascular risk. However, in OSA patients without cardiovascular disease neither MADs nor CPAP significantly affected left ventricular outcomes after 2–3 months [27]. These findings suggest that longer-term follow-up is necessary to fully assess the effects of OSA treatments on myocardial function. Additionally, the lack of immediate changes in LV structure in this study underscores the complexity of cardiac remodeling in OSA and suggests that individual patient factors, such as the presence of underlying cardiac conditions, may influence treatment options and outcomes. Further insights into the impact of MAD therapy on myocardial function are supported by animal studies. Untreated OSA-induced rabbits showed severe myocardial damage, while MAD-treated ones did not, suggesting that early intervention with MADs might prevent myocardial damage, potentially preventing fibrosis and hypertrophy [16].

N-terminal pro-brain natriuretic peptide (NT-proBNP) is an important biomarker for assessing cardiovascular risk, especially in patients with heart failure and left ventricular dysfunction. Elevated NT-proBNP concentrations are associated with increased cardiac stress., making it usefull for assessing OSA treatment efficacy. In OSA patients without pre-existing cardiovascular disease, MADs reduced the NT-proBNP concentration, suggesting that it might alleviate the cardiac strain associated with OSA, whereas CPAP showed no significant improvement, with NT-proBNP levels slightly increasing, implying MADs might better reduce subclinical cardiovascular stress in certain patients [27]. On the other hand, a longer-term study in moderate OSA patients found no significant NT-proBNP change with either treatment, possibly it could be attributed to a younger and healthier study population with minimal cardiac dysfunction [28]. This highlights the potential for variability in NT-proBNP response, depending on patient demographics and baseline cardiovascular health. Furthermore, the contrasting findings between these studies suggest that NT-proBNP may be a more sensitive marker in OSA patients with elevated cardiovascular risk or early signs of cardiac stress. These discrepancies highlight the need for large-scale studies on individuals at high CVD risk to assess the effects of OSA treatment using either MADs or CPAP, potentially guiding more personalized therapeutic approaches.

Cardiovascular events, including stroke, myocardial infarction, and sudden cardiac arrest are strongly associated with untreated or inadequately treated OSA, making them critical endpoints when evaluating the effectiveness of MAD treatment [36]. While CPAP was found to be more effective in reducing the AHI, patient compliance was significantly higher with MAD therapy [17]. As a result, the overall health outcomes, including 24-hour mean arterial blood pressure and quality of life, were similar between the two treatments [24]. This highlights the importance of adherence in OSA therapy: while CPAP might provide superior efficacy in reducing AHI, its lower compliance rate diminishes its overall cardiovascular benefit relative to MADs. The comparable CV results suggest that MADs can offer an effective alternative for patients who struggle with CPAP, particularly when long-term cardiovascular health is a priority. A large-scale randomized trial in severe OSA patients treated with CPAP or MADs revealed both therapies similarly reduced cardiovascular mortality risk, with untreated patients showing a significantly higher incidence of cardiovascular death [30]. This underscores the life-saving potential of OSA treatment, highlighting the importance of adherence.

These findings emphasize that while the reduction in AHI is a primary measure of OSA treatment success, cardiovascular outcomes are equally important given the high prevalence of cardiovascular disease in OSA patients. The similarity in CV risk reduction between CPAP and MAD therapy suggests that adherence to treatment plays a critical role in achieving cardiovascular benefits. MADs, despite slightly less effective in AHI reduction, often have higher compliance, leading to consistent usage that might compensate for this difference and result in comparable cardiovascular outcomes. In summary, the treatment of OSA with MADs targets multiple pathophysiological mechanisms that are closely linked to cardiovascular risk. Through the modulation of heart rate variability, MAD therapy improves autonomic balance, which is crucial for cardiovascular health and reduces the risk of morbidity and mortality. Additionally, MAD therapy has been shown to positively impact left ventricular function and myocardial remodeling, with some evidence suggesting a reduction in interventricular septal thickness and mitigation of hypertrophic changes, thereby offering cardioprotective benefits similar to those observed with CPAP. MADs also play a role in reducing oxidative stress and inflammation, both of which are key contributors to endothelial dysfunction in OSA. Improvements in EF and decreases in oxidative stress markers highlight the ability of MADs to restore vascular health in OSA patients. Furthermore, reductions in NT-proBNP levels following MAD therapy indicate a decrease in cardiac strain, suggesting that MADs lower the risk of long-term cardiovascular complications such as heart failure. However, it is important to consider NT-proBNP alongside other clinical factors, as its response to therapy may vary depending on the patient’s baseline cardiovascular condition. Finally, the reduction in cardiovascular events, including fatal events, underscores the significance of treating OSA with MADs. While CPAP may offer superior efficacy in reducing AHI, the higher adherence rates observed with MADs make them a valuable alternative for achieving comparable cardiovascular outcomes.

## 4. Limitations

In addition to the strengths of our research, several limitations should be acknowledged. Firstly, the variability in individual patient responses to MAD therapy, particularly regarding its effects on cardiovascular outcomes like endothelial function and oxidative stress, suggests that patient-specific factors may influence treatment efficacy. One key limitation is the inconsistent effect of MAD therapy on nitric oxide levels. Although some studies indicate an increase in NO concentrations with MAD treatment, this effect has not been consistently observed across all patient groups, suggesting that the ability of MADs to enhance endothelial function may depend on individual patient characteristics, such as OSA severity, baseline cardiovascular health, or genetic factors. Additionally, while MADs show promise in improving NO-mediated vasodilation, the mechanisms underlying these variations remain poorly understood, and the long-term cardiovascular benefits of MAD therapy in this context are still uncertain. This highlights the need for further research to elucidate the factors influencing NO response and its clinical relevance in reducing cardiovascular risk in OSA patients. Additionally, while CPAP is more effective in reducing AHI, the comparative cardiovascular benefits of MADs are primarily based on higher adherence rates, which might not apply universally. Furthermore, the long-term impact of MAD therapy on cardiac remodeling and biomarkers like NT-proBNP remains unclear, necessitating more extensive, longitudinal studies. Finally, most studies focus on relatively small cohorts, limiting the generalizability of their findings.

## 5. Conclusions

In conclusion, MAD therapy addresses multiple cardiovascular mechanisms—oxidative stress, inflammation, endothelial dysfunction, NT-proBNP concentrations, LV function, HRV, and the prevention of cardiovascular events—making it an essential treatment option for reducing the cardiovascular risk associated with OSA. Further research is needed to explore long-term outcomes and refine patient selection to maximize the cardioprotective effects of MADs.

## 6. Future Directions

Critical future direction lies in the development of personalized treatment strategies for OSA. Currently, the choice among CPAP, MAD, and surgical options is often made based on the severity of OSA and patient preferences. Personalized medicine tailored to the specific characteristics of each patient could significantly improve patients’ outcomes. Collaborations between sleep specialists, cardiologists, and primary care physicians will be vital in optimizing treatment for OSA patients with high cardiovascular risk. By working together to develop comprehensive treatment plans, healthcare providers can ensure that patients receive the most effective and individualized care possible. Future research should focus on long-term outcomes, personalized treatment pathways and the integration of cardiovascular biomarkers to guide therapy decisions. These efforts will help ensure that each patient receives the treatment best suited to their unique needs, ultimately improving not only their sleep but also their cardiovascular health.

## Figures and Tables

**Table 1:** Effects of mandibular advancement device therapy on oxidative stress.

Author, Year	N	Sex	Age, Years	AHI (Pre)	AHI (Post)	Biomarker (Pre)	Biomarker (Post)
Itzhaki et al., 2007 [14]	16	11 M/ 5 F	54.0 ± 8.3	29.7 ± 18.5 *	17.7 ± 11.1 * (3 mo); 19.6 ± 11.5 * (1 y)	TBARS: 18.8 ± 6.2	TBARS: 15.8 ± 3.9 (3 mo) **; 15.5 ± 3.2 (1 y) ***
Lin et al., 2014 [15]	C: 15	12 M/3 F	48 ± 7	3.2 ± 0.9	N/D	NOx: N/D	NOx: N/D
	S: 19MADs	24 M/6 F	50 ± 8	31.5 ± 11.4	8.6 ± 5.7 ^	11.8 ± 5.8 ***	22.7 ± 4.9 ***
	S: 11MADf		51 ± 7	31.8 ± 11.6	24.7 ± 5.9	N/D	N/D
Liu et al., 2020 [16]	control group: 10	10 rM	36-month-old	N/D	N/D	ET-1: N/D Ang II: N/D ET-1 mRNA: N/D	ET-1: N/D Ang II: N/D ET-1 mRNA: N/D
	OSA group: 10	10 rM	36-month-old	N/D	N/D	ET-1: N/D Ang II: N/D ET-1 mRNA: N/D	ET-1: N/D Ang II: N/D ET-1 mRNA: N/D
	MAD group: 10	10 rM	36-month-old	N/D	N/D	ET-1: N/D Ang II: N/D ET-1 mRNA: N/D	ET-1: N/D Ang II: N/D ET-1 mRNA: N/D
Dal-Fabbro et al., 2014 [17]	29	24 M/5 F	47.0 ± 8.9	42.3 ± 24.3	25.0 ± 12.4 ^	TBARS: 1.1 ± 0.2 catalase: 57.9 ± 2.6 superoxide dismutase: 5.7 ± 0.7 vitamins: - C: 54.9 ± 2.5 - E: N/D - B6: 18.9 ± 1.2 - B12: N/D folate: N/D homocysteine:N/D uric acid: N/D	TBARS: POA: 1.3 ± 0.2 MAD: 1.4 ± 0.2 CPAP: 1.4 ± 0.1 catalase: POA: 51.3 ± 2.2 MAD:47.6 ± 2.3 ^ CPAP: 53.6 ± 1.9 superoxide dismutase: POA: 5.2 ± 0.6 MAD: 5.2 ± 0.7 CPAP: 4.8 ± 0.6 vitamins: - C: POA: 65.9 ± 3.0 ^ MAD: 62.3 ± 2.6 CPAP: 64.3 ± 2.5 ^ - E: N/D - B6: POA: 22.7 ± 1.5 ^ MAD: 22.2 ± 1.3 ^ CPAP: 24.6 ± 0.9 ^ - B12: N/D folate: N/D homocysteine: N/D uric acid: N/D

Abbreviations: M, men; F, female; AHI, apnea-hypopnea index; rM, rabbit male; C, control group; S, study group; *, *p* < 0.005; **, *p* = 0.09; ***, *p* < 0.05; ^; *p* < 0.05; MAD_f_, mandibular advancement device therapy failure; MAD_s_, mandibular advancement device therapy successful; 3 mo, 3 months follow-up; 1 y, 1-year follow-up; ET-1, endothelin-1; Ang-2, Angiotensin II; OSA, obstructive sleep apnea group; MAD, mandibular advancement device group; POA, placebo oral appliance group; TBARS, thiobarbituric acid-reactive substances; NOx, nitric oxide derivatives; N/D, no data.

**Table 2:** Effects of mandibular advancement device therapy on inflammation.

Author, Year	N	Sex	Age, Years	AHI (Pre)	AHI (Post)	Biomarker (Pre)	Biomarker (Post)
Kang et al. 2024 [18]	6	6 rM	6 month-old	N/D	N/D	NF-κB: N/D	NF-κB: N/D
	6	6 rM	6 month-old	N/D	N/D	NF-κB: N/D	NF-κB: N/D
	6	6 rM	6 month-old	N/D	N/D	NF-κB N/D	NF-κB: N/D
Niżankowska-Jędrzejczyk et al. 2014 [19]	C:16	16 rM	54.06 ± 12.09	2.05 (1.13–3.55)	N/D	IL-1β: 0.24 (0.21–0.27) IL-10: 3.93 (3.13–4.86)	IL-1β: 0.2—(0.16–0.24) 6 mo IL-10: 3.91 (2.88–499) 6 mo
	MAD:22	22 rM	52.5 ± 8.33	24 (15.7–31.25)	13.1 (4.98–21.4) 3 mo 7.05 (4.3–11.65) 6 mo	IL-1β: 0.35 (0.21–0.39) * IL-10: 5.71 (2.83–7.88)	IL-1β: - 3 mo: 0.19 (0.18–0.25) *** - 6 mo: 0.22 (0.19–0.25) ** IL-10: - 3 mo: 3.27 (2.26–5.12) - 6 mo: 2.95 (2.09–4.06)

Abbreviations: M, men; F, female; AHI, apnea-hypopnea index; rM, rabbit male; c, control group; s, study group; 6 mo, 6 months follow-up; 3 mo, 3 months follow-up; NF-κB, nuclear factor-kappa B; IL-1β, interleukin-1β; IL-10, interleukin-10; C, control group; MAD, study group treated with mandibular advanced devices; *, *p* < 0.05 significant difference between baseline control and baseline OSA group; **, *p* < 0.05; ***, *p* < 0.01 significant difference between baseline and 3 month treatment for OSA group.

**Table 3:** Effects of mandibular advancement device therapy on endothelial function.

Author, Year	N	Sex	Age, Years	AHI (Pre)	AHI (Post)	Endothelium Function	
						(Pre)	(Post)
Itzhaki et al., 2007 [14]	S: 16	11 M/ 5 F	54.0 ± 8.3	29.7 ± 18.5 *	17.7 ± 11.1 * (3 mo); 19.6 ± 11.5 * (1 y)	RH-PAT index: 1.77 ± 0.4	RH-PAT index: - 3 mo: 2.1 ± 0.4 *** - 1 y: 2.0 ± 0.3 #
	C: 6	4 M/2 F	42.7 ± 11.2	31.0 ± 8.1	29.2 ± 8.9 (1 y)	1.9 ± 0.4	1.7 ± 0.4
Lin et al., 2014 [15]	C: 15	12 M/3 F	48 ± 7	3.2 ± 0.9	N/D	N/D	
	S: 19MADs	24 M/6 F	50 ± 8	31.5 ± 11.4	8.6 ± 5.7 ***	FMD: 5.9 ± 4.6	FMD: 10.5 ± 4.8 ***
	S: 11MADf		51 ± 7	31.8 ± 11.6	24.7 ± 5.9	no significant improvement	
Gagnadoux et al. 2017 [20]	S: 75	59 M/16 F	54.8 ± 9.9	40 (34–50.5)	18.5 ** (11.5–26)	RHI: 2.13 ± 0.6	RHI: 2.1 ± 0.63
	P: 75	70 M/5 F	52.9 ± 10.5	47.0 (36.0–58.0)	38 (23–51) **	2.17 ± 0.63	2.04 ± 0.59
Trzepizur et al. 2009 [21]	CPAP: 6	N/D	56 (56–58)	40 (31–49)	2 (1–8) ***	Ach: 1.9 (1.7–2.7)	Ach: 2.6 (2.3–4.4) ***
	MAD: 6	N/D			14 (7–18) ***		2.7 (1.9–4.3) ***

Abbreviations: M, men; F, female; AHI, apnea-hypopnea index; rM, rabbit male; C, control group; S, study group; P, placebo; *, *p* < 0.005; **, *p* < 0.01; ***, *p* < 0.05; #, *p* = 0.055; MAD, mandibular advancement device group; MADf, mandibular advancement device therapy failure; MADs, mandibular advancement device therapy successful; 3 mo, 3 months follow-up; 1 y, 1-year follow-up; CPAP, continuous positive airway pressure; RH-PAT, reactive hyperemia peripheral arterial tonometry; FMD, flow-mediated dilation; RHI, reactive hyperemia index; Ach, acetylcholine; N/D, no data.

**Table 4:** Effects of mandibular advancement device therapy on heart rate variability.

Author, Year	N	Sex	Age, Years	AHI Pre	AHI Post	Biomarker Pre	Biomarker Post
Kim et al. 2020 [22]	58	51M/7F	N/D	41.0 ± 20.1	19.6 ± 17.1 *	- average NN interval: 949.3 ± 134.1 - HFnu: 29.5 ± 11.9 - LFnu: 70.5 ± 11.9 - NN50 count: 3089 ± 3175 - Total power: 51,905 ± 25,730 - VLF: 29,058 ± 18,160 - LF: 15,884 ± 7984 - HF: 5790 ± 2619 - LF/HF ratio: 3.0 ± 1.9	- average NN interval: 988.4 ± 127.0 ** - HFnu: 32.6 ± 13.8 *** - LFnu: 67.4 ± 13.8 *** - NN50 count: 2860 ± 2944 - Total power: 48,241 ± 25,666 - VLF: 26,480 ± 17,101 - LF: 14,929 ± 9359 - HF: 6140 ± 2720 - LF/HF ratio: 2.8 ± 2.0
Glos et al. 2016 [23]	CPAP:21 MAD:19	N/D	N/D	N/D	N/D	HRV: - HF: CPAP: 15.9 ± 22.7 MAD: 15.9 ± 22.7 BRS: - α-LF: CPAP: 7.1 ± 4.0 MAD: 7.1 ± 4.0	HRV: - HF: CPAP: 19.0 ± 31.5 MAD: 22.3 ± 30.3 BRS: - α-LF: CPAP: 6.4 ± 2.8 MAD: 6.7 ± 3.8
Lee et al. 2020 [24]	CPAP: 30 MAD: 30	27M/3F 27M/3F	42.5 ± 11.8 51.6 ± 9.2	44.7 ± 25.9 44.0 ± 20.3	21.9 ± 20.1 * 21.3 ± 19.5 *	- average NN interval: 942.2 ± 140.8 (s) 901.1 ± 131.7 (m) - LF: 16,812 ± 10,531 (s) 15,590 ± 7962 (m) - HF: 7083 ± 3963 (s) 5743 ± 2755 (m) - LF/HF ratio: 2.9 ± 1.8 (s) 3.0 ± 1.8 (m) - LFnu: 69.0 ± 13.2 (s) 70.9 ± 10.7 (m) - HFnu: 31.0 ± 13.2 (s) 29.1 ± 10.7 (m)	- average NN interval: 994.6 ± 143.1 (s) *** 953.7 ± 123.1 (m) *** - LF: 13,710 ± 8381(s) *** 13,599 ± 8906 (m) - HF: 7348 ± 3517 (s) 6087 ± 2625 (m) - LF/HF ratio: 2.3 ± 1.7 (s) *** 2.4 ± 1.4 (m) - LFnu: 63.2 ± 13.7 (s) *** 66.3 ± 12.5 (m) *** - HFnu: 36.8 ± 13.7 (s) *** 33.7 ± 12.5 (m) ***
Dal-Fabbro et al. 2014 [17]	29	24M/5F	47.0 ± 8.9	42.3 ± 24.3	25.0 ± 12.4^	Baseline HRV: - Total power (night): 17,536.2 ± 1561.2 - LF(night): 4814.4 ± 365.7 - HF(night): 1556.3 ± 167.4 - ISAV: 2854.9 ± 580.0	POA HRV: - Total power (night): 19,108.8 ± 1759.1 - LF(night): 4939.2 ± 338.7 - HF(night): 1545.8 ± 203.0 - ISAV: 1823.8 ± 500.9 MAD HRV: - Total power(night): 15,576.9 ± 1123.3† - LF(night): 4354.4 ± 363.8 - HF(night): 1581.2 ± 226.0 - ISAV: 1491.0 ± 328.6 *** CPAP HRV: - Total power(night): 14,608.6 ± 960.9 *** - LF(night): 4499.5 ± 391.1 - HF(night): 1098.8 ± 104.3 *** - ISAV: 1883.1 ± 406.1
Ucak et al. 2024 [25]	101	54M/47F	56	27 (17)	10 (11) *	average NN: 982 (173) SDNN: 44 (27) RMSSD: 34 (24) pNN50: 12 (24) TP: 2237 (2651) LF: 2607 (998) HF: 494 (698) LF/HF: 1.2 (1.3) LFnu: 51 (17) HFnu: 42 (15)	average NN: 1017 (181) SDNN: 42 (31) *** RMSSD: 31 (24) *** pNN50: 9 (22) TP: 1991 (2951) *** LF: 559 (1186) HF: 355 (635) *** LF/HF: 1.4 (1.4) *** LFnu: 54 (18) *** HFnu: 39 (16) ***

Abbreviations: *, *p* < 0.001; **, *p* = 0.001; ***, *p* < 0.05; (SD), standard deviation; AHI apnea-hypopnea index; CPAP, continuous positive airway pressure group; HRV, heart rate variability; BRS, baroreceptor sensitivity; s, surgery group; m or MAD, mandibualr advancement device group; ISAV, index of sleep autonomic variation; POA, placebo oral appliance group; SDNN, standard deviation of NN intervals; RMSSD, the square root of the mean of the squared differences of adjacent NN intervals; NN50 count, number of pairs of adjacent NN intervals more than 50 ms; pNN50, rate of NN50 in the total number of NN intervals; VLF, very low frequency; LF, low frequency; HF, high frequency; LFnu, LF power in normalized units; HFnu, HF power in normalized units; TP, total power.

**Table 5:** Effects of mandibular advancement device therapy on left ventricle structure and function.

Author, Year	N	Sex	Age, Years	AHI Pre	AHI Post	Echographic Parameters (Pre)	Echographic Parameters (Post)
Dieltjens et al. 2022 [26]	63	45M/18F	49 ± 11	11.7 [8.2; 24.9]	5.6 [2.9; 13.1] *	LV geometry and LV mass: - IVS thickness 11.1 ± 2.1 - LVIDd 47.9 ± 6.0 - LVIDs 31.3 ± 5.2 - PW thickness 8.8 ± 1.7 - LV mass 173.9 ± 51.6 LV systolic function - LVEF 60.3 ± 6.1 - TDI med s’ 7.4 ± 1.5 - TDI lat s’ 9.1 ± 2.1 LV diastolic function - E/A 1.0 ± 0.3 - E/e’ 9.3 ± 2.3 - LA vol index 29.4 ± 7.6	LV geometry and LV mass - IVS thickness 10.6 ± 2.0 - LVIDd 47.8 ± 5.6 - LVIDs 30.8 ± 4.0 - PW thickness 8.6 ± 2.7 - LV mass 160.5 ± 43.3 * LV systolic function - LVEF 60.8 ± 6.4 - TDI med s’ 7.6 ± 1.7 - TDI lat s’ 9.0 ± 2.3 LV diastolic function - E/A 1.0 ± 0.3 - E/e’ 9.3 ± 2.3 - LA vol index 29.9 ± 8.3
Hoekema et al. 2008 [27]	CPAP: 13 MAD: 15	25M/3F	49.7 ± 9.9	CPAP: 54.8 [50.9–73.7] MAD: 31.7 [23.9–36.5]	CPAP: 2.0 [0.0–6.3] MAD: 2.4 [0.9–10.2]	MAD: LVPW (*n =* 8) 9.1 ± 1.6 LVIVS (*n =* 8) 9.8 ± 1.3 LVEDD (*n* = 9) 49.1 ± 3.2 LVESD (*n* = 9) 30.8 ± 4.7 LVMI (*n* = 8) 89.5 ± 22.0 LVEF *n* = 10 56.9 ± 3.8 CPAP: LVPW (*n* = 11) 10.5 ± 1.0 LVIVS (*n* = 11) 10.7 ± 1.6 LVEDD (*n* = 11) 48.3 ± 6.4 LVESD (*n* = 11) 28.2 ± 4.1 LVMI (*n* = 11) 96.5 ± 28.9 LVEF *n* = 9 58.8 ± 2.3	MAD: LVPW (*n* = 8) 10.1 ± 0.8 LVIVS (*n* = 8) 9.9 ± 1.0 LVEDD (*n* = 9) 48.0 ± 4.9 LVESD (*n* = 9) 29.9 ± 4.7 LVMI (*n* = 8) 91.3 ± 18.7 LVEF *n* = 10 60.5 ± 5.3 CPAP: LVPW (*n* = 11) 10.5 ± 1.9 LVIVS (*n* = 11) 11.1 ± 2.0 LVEDD (*n* = 11) 47.5 ± 4.3 LVESD (*n* = 11) 28.7 ± 5.5 LVMI (*n* = 11) 98.1 ± 28.9 LVEF *n* = 10 59.7 ± 1.3
Liu et al. 2020 [16]	control group: 10	10rM	36-month-old	N/D	N/D	N/D	N/D
	OSA:10	10rM	36-month-old	N/D	N/D	N/D	- decreased E/*a*’ ratio - increased E/*e*’ ratio - decreased LVEF
	MAD: 10	10rM	36-month-old	N/D	N/D	N/D	- no significant difference compared to MAD group

Abbreviations: [Quartile 1; Quartile 3], values presented as median; *, *p* < 0.05; IVS, interventricular septum thickness; LA vol index, left atrium indexed volume; LV, left ventricle; LVEF, left ventricular ejection fraction; LVIDd, left ventricular internal diameter end diastole; LVIDs, left ventricular internal diameter end systole; PW, posterior wall thickness; TDI lat, tissue doppler imaging lateral wall; TDI med, tissue doppler imaging medial wall; e′, tissue Doppler derived early diastolic velocity; s′, tissue Doppler derived systolic velocity; CPAP, continuous positive airway pressure group; MAD, mandibular advancement device group; LVEDD, left ventricular end-diastolic diameter; LVEF, left ventricular ejection fraction; LVESD, left ventricular end-systolic diameter; LVIVS, diastolic interventricular septal thickness; LVMI, left ventricular mass index; LVPW, left ventricular posterior wall diastolic thickness; OSA, obstructive sleep apnea group; rM, male rabbits; N/D—no data; *E*, the peak of early diastolic velocities; *A*, the peak of late diastolic velocities; *a*’, late diastolic mitral annulus velocities; E/*a*’ ratio, an estimate of left ventricular diastolic function; *e*’, early diastolic mitral annulus velocities; E/*e*’ ratio, an estimate of LV end-diastolic pressures; AHI, apnea-hypopnea index.

**Table 6:** Effects of mandibular advancement device therapy on NT-proBNP.

Author, Year	N	Sex	Age, Years	AHI Pre	AHI Post	NT-proBNP (Pre)	NT-proBNP (Post)
Hoekema et al. 2008 [27]	28 CPAP:13 MAD:15	25 M/ 3F	49.7 ± 9.9	CPAP: 54.8 (50.9–73.7) MAD: 31.7 (23.9–36.5)	CPAP: 2.0 (0.0–6.3) MAD: 2.4 (0.9–10.2)	CPAP: 31 (17–53) MAD: 52 (13–105)	CPAP: 37 (21–61) MAD: 22 (15–33)
Uniken Venema et al. 2022 [28]	85 MAD:43 CPAP:42	37 M/6 F 33 M/9 F	50 ± 9.7 51 ± 9.8	20.9 ± 4.4 21.0 ± 4.7	10.8 ± 11.9 *#^ 1.5 ± 1.6 *#^	24.2 (12.3–57.8) 39.3 (17.3–61.3)	26.0 (11.3–52.0) 31.4 (12.2–56.8)

Abbreviations: values with additives in parenthesis are medians with interquartile ranges; CPAP, continuous positive airway pressure group; MAD, mandibular advancement device group; M, male; F, female; AHI, apnea-hypopnea index; NT-proBNP, N-terminal pro–hormone B-type natriuretic peptide; *, significant difference between MAD and CPAP at baseline or follow-up; #, significant difference in delta change from baseline to 12-months between MAD and CPAP, ^, significant difference between baseline and follow-up.

**Table 7:** Effects of mandibular advancement device therapy on cardiovascual events.

Author, Year	N	Sex	Age, Years	AHI Pre	AHI Post	Cardiovascular Events
Philips et al. 2013 [29]	126	102 M/24 F	49.5 (11.2)	25.6 (12.3)	CPAP: 4.5 (6.6) # MAD: 11.1 (12.1) #	N/D
Anandam et al. 2013 [30]	Non-apnoeic controls: 208 Severe OSA treated with CPAP: 177 Severe OSA treated with MAD: 72 Untreated severe OSA: 212	113 M/95 F 108 M/69 F 41 M/31 F 138 M/74 F	46.8 ± 13.7 51.6 ± 12.3 50.8 ± 12.7 50.3 ± 13.2	2.6 ± 1.4 44.8 ± 9.4 * 44.5 ± 7.7 ** 43.4 ± 8.6 ***	N/D N/D N/D N/D	Cardiovascular death/events per 100 person-years: 4/0.28 6/0.56 † 3/0.61 ‡ 29/2.1 §

Abbreviations: OSA, obstructive sleep apnoea; MAD, mandibular advancement device; CPAP, continuous positive airway pressure; M, men; F, female; *, *p* < 0.05 for comparisons of severe OSA treated with CPAP and non-apnoeic controls; **, *p* < 0.05 for comparisons of severe OSA treated with MAD and non-apnoeic controls; ***, *p* < 0.05 for comparisons of untreated severe OSA and non-apnoeic controls; †, *p* < 0.001 compared to the non-apnoeic controls; ‡, *p* < 0.05 compared to untreated severe OSA; §, *p* < 0.001 compared to untreated severe OSA; #, *p* < 0.001.; N/D, no data.

## Data Availability

Not applicable.
